# Cold Feet

**Published:** 1874-01

**Authors:** 


### COLD FEET.

It is hurtful, even dangerous to go to bed with feet as "cold as ice;" which is often the case, even with persons in good health, who have been on their feet all day, or have walked a great deal without having taken off their shoes or boots, because the perspiration has condensed and made the feet damp; you feel them with your hand and there is the cold clamminess of the grave; there is not blood enough there to keep them warm; the excess has retreated to the brain, to over excite it, and you can't sleep.

One of the most comfortable and healthful habits in the world, during the whole fire time of year, is to pull off both shoes and stockings the last thing before going to bed, and hold the feet to the open fireplace, or grate, or register, or to the stove or range in the kitchen, rubbing the soles all the time with the hands, until they are perfectly warmed, and not an atom of dampness is perceived between the toes or anywhere else; when this is done draw on your stockings or slippers, wash your hands, and get into bed, for if you walk on the cold floor the feet may be chilled again.

If the feet are inclined to become cool after you get in bed, considerable benefit is derived by wrapping each foot in a newspaper, because it is so compact that the warm air cannot readily escape from within.

Some persons require so much bed clothing, especially from above the knees downwards, that it is an oppressive weight, and prevents sleep; a good plan in such cases is to get three large newspapers—one is better than none—spread them out, make a binding on the outer edges, and make a cross with long stitches from side to side, to keep the three in place, and to prevent tearing at the edges; there is as much warmth in these, if thrown over the lower limbs, as in a thick blanket, while it is not near so heavy; the philosophy of it being that the impervious paper keeps the natural warmth of the body in and around the limbs better than the blanket, which is full of little holes.

But there is a large number of persons, especially in cities—unfortunate old bachelors and young clerks, and others—who have no cheerful, warm sitting room to go home to, when night comes, who cannot afford to have a fire to go to bed by, and who, often, from one cause or another, are conscious of having cold, clammy, damp feet, as they get into bed. If hot water cannot be had, take a towel and wipe the feet as perfectly dry as possible, then patiently rub them with the palms of the hands until they are somewhat warmed, and then either draw on thick woolen stockings, which may be taken off later in the night, or wrap the extremities up in a newspaper.

Persons who sit or stand a greater part of the day at their places of business should be very careful, on first entering, to wipe the soles of their shoes as dry as possible, hold them to the fire a few minutes, and have a thick woollen mat to rest them on. Many persons have contracted a habit of cold feet by standing on wooden floors or benches all day; the bench is worse than the floor, because there is a cold current of air passing between it and the floor.

These may appear to be trivial things to many; but nothing is trivial which prevents disease or promotes health, for without that we are worthless to ourselves and a burden to others; there is no joy or gladness in the blue sky or in the sunshine, and fine houses, and lovely families, and admiring friends, and millions of money avail not to mitigate a single pain; if anything, they but aggravate the malady.

